# Pan-neurofascin autoimmune nodoparanodopathy: A case report and literature review

**DOI:** 10.1097/MD.0000000000041304

**Published:** 2025-01-24

**Authors:** Elsa Krim, Alexandre Masri, Emilien Delmont, Gwendal Le Masson, Joseph Boucraut, Stéphane Mathis

**Affiliations:** a Department of Neurology, Pau Hospital, Pau, France; b Department of Reanimation, Pau Hospital, Pau, France; c Department of Neurology (Nerve-Muscle Unit), Reference Center for Neuromuscular Diseases, University Hospitals of Marseille, Marseille, France; d Department of Neurology (Nerve-Muscle Unit), Reference Center for Neuromuscular Diseases “AOC,” ALS Reference Center, University Hospitals of Bordeaux (Pellegrin Hospital), University of Bordeaux, Bordeaux, France; e Department of Immunology, INS Institut de Neurosciences des Systèmes – INSERM U 1106 (Physionet Team), University Hospitals of Marseille, Marseille, France.

**Keywords:** case report, locked-in syndrome, pan-neurofascin, IgG4, rituximab

## Abstract

**Rationale:**

Locked-in syndrome (and its variant, completely locked-in state) generally has a high mortality rate in the acute setting; however, when induced by conditions such as acute inflammatory polyradiculoneuropathy, it may well be curable such that an attempt at cure should be systematically sought by clinicians.

**Patient concerns:**

A 52-year-old man presented with acute tetraparesia and areflexia, initially diagnosed as Guillain–Barré syndrome. Despite appropriate treatment, his condition deteriorated, evolving into a completely locked-in state.

**Diagnoses:**

The detection of anti-pan-neurofascin antibodies led to the correct diagnosis, acute pan-neurofascin autoimmune nodoparanodopathy.

**Interventions:**

Immunosuppressive treatment (rituximab) and plasma exchanges were performed.

**Outcomes:**

After several months, the patient’s neurological symptoms almost completely subsided, without any major sequelae.

**Lessons:**

In patients with locked-in syndrome (or its variant), neurologists and intensive care physicians must be aware of, and look for, the main etiologies (including pan-neurofascin autoimmune nodoparanodopathy), to allow the prompt initiation of treatment and thus a rapid recovery for these ultimately curable conditions. Despite causing major disability, pan-neurofascin autoimmune nodoparanodopathy is curable if the appropriate treatment is given.

## 1. Introduction

Guillain–Barré syndrome (GBS) is an inflammatory polyneuropathy distinguished by a gradual onset of symmetrical, flaccid hypoxia in the ascending extremities, accompanied by motor symptoms with or without sensory manifestations. However, it encompasses several distinct clinical phenotypes apart from the classic form. Prototypical GBS consists of an acute inflammatory demyelinating polyradiculoneuropathy (AIDP), including generalized axonal subtypes, such as acute motor axonal neuropathy (AMAN), and acute motor and sensory axonal neuropathy (AMSAN), both of which are nodopathies, as well as regional variants, such as Miller Fisher syndrome, pharyngeal-cervical-brachial syndrome, and nodoparanodopathies. Autoimmune neuropathies constitute a large spectrum of disorders, usually classified on the basis of their clinical, pathological, and electrophysiological features as acute polyradiculoneuropathies, the classical primary demyelinating condition referred to as GBS, or a primary axonal condition (AMAN or AMSAN). More recently, immune-mediated neuropathies have been classified with reference to the involved domains of the myelinated fibers and (when known) the target antigens, exemplified by internodopathies (such as in acute inflammatory demyelinating polyradiculoneuropathy) and nodoparanodopathies (such as AMAN/AMSAN).^[1,2]^ The myelinated fibers are organized in distinct domains (node, paranode, juxta-paranode and inter-node), with the node of Ranvier (NoR) a common site of attack in inflammatory neuropathies. The term “nodoparanodopathy” was originally proposed to characterize more accurately neuropathies associated with anti-ganglioside antibodies and a common pathogenic mechanism of dysfunction/disruption at the NoR, resulting in a pathophysiological continuum from transitory nerve conduction failure to axonal degeneration.

In the following, we present a patient with locked-in syndrome associated with an acute pan-neurofascin autoimmune nodoparanodopathy. Prompt treatment led to a favorable outcome.

## 2. Case presentation

**Figure 1.**
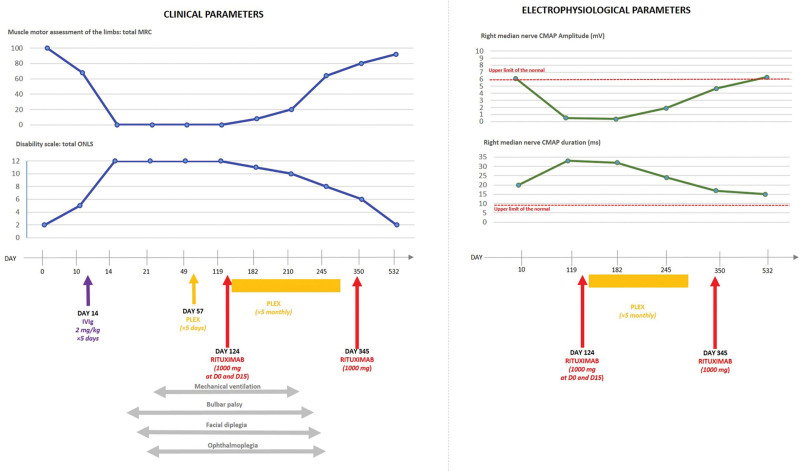
Schematic of the main clinical course and the electrophysiological parameters during the 532-day follow-up of our patient. The muscle motor assessments of the 4 limbs were evaluated by deriving the MRC scores (normal muscle force = 100). Disability was evaluated using the total overall neuropathy limitations scale (ONLS; maximum score = 12; 0, no disability; 12, total disability). In the electrophysiological context, we showed the evolution of the CMAP amplitude (an “axonal parameter”) and the duration thereof (a “demyelinating” parameter) for the right median nerve (at wrist) compared to the normal values represented by the dotted red line. CMAP = compound muscle action potential, IVIg = intravenous immunoglobulin, mg = milligram, ms = millisecond, mV = millivolt, PLEX = plasma exchange.

**Figure 2.**
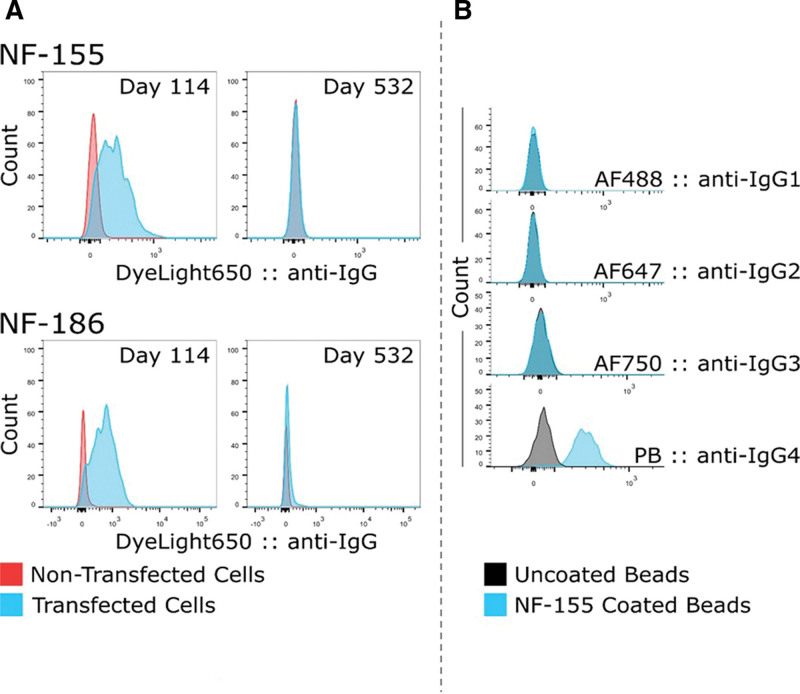
Sera (diluted 1:100) obtained before and after treatment were incubated with cells transfected to allow NF155 and NF186 expression (A) or beads with recombinant NF155 (B). After washing, cells and beads were incubated with anti-IgG (A), anti-IgG1, anti-IgG2, anti-IgG3, or anti-IgG4 fluorescence-labeled antiserum (B). The fluorescence levels were compared to those obtained using non-transfected cells or control beads. We observed a positive result for anti-NF155 and anti-NF186 antibodies at D114, with negativity at D532 (A); these were only anti-IgG4 antibodies (B). AF-488 = Alexa FluorTM 642 (it specifically detects IgG1 antibodies), AF-642 = Alexa FluorTM 642 (it specifically detects IgG2 antibodies), AF-750 = Alexa FluorTM 750 (it specifically detects IgG3 antibodies), NF = neurofascin, PB = plasmablasts (circulating plasmablasts are elevated in active IgG4 related diseases, so it is used as a biomarker of IgG4 antibodies).

A 52-year-old man with no particular medical history developed paresthesia of the hands and feet within a few hours, followed by distal motor weakness in all 4 limbs and generalized areflexia within a few days. No infection or recent animal bites were found. At day 10 (D10), he was admitted to a hospital. An electrophysiological study (electroneuromyography; Table 1) revealed a sensorimotor demyelinating polyneuropathy with prolonged distal motor latencies in all 4 limbs, decreased compound muscle action potential (CMAP) amplitudes in the lower limbs and right ulnar nerve, and moderately decreased sensory nerve action potentials in all 4 limbs. His spine magnetic resonance imaging results were normal. Lumbar puncture revealed mild elevation of the protein level in the cerebrospinal fluid (CSF; 47 g/dL; normal < 40 g/dL), but no cells. Anti-ganglioside and anti-neuronal autoantibody assays were negative in both serum and the CSF. The remaining blood work-up was normal. Following a tentative diagnosis of GBS, intravenous immunoglobulin (IgIV; 2 mg/kg over 5 days) was initiated on D11 (D10: Guillain–Barré syndrome Disability Scale [GBS-DS] of 3/6; total modified ERASMUS GBS outcome score at D7 of 4). Five days later, despite treatment, the patient was tetraplegic and developed dysarthria and autonomic dysfunction: in 5 days, he successively presented sinusal tachycardia (100–120/minute), then 3 episodes of bradycardia (35–45/minute), then 2 episodes of arterial hypotension, then 2 episodes of bradycardia (35–45/minute). He was admitted to the medical intensive care unit wherein he developed facial diplegia and complete ophthalmoplegia in addition to requiring mechanical ventilation and gastrostomy (Fig. 1; D15: GBS-DS score of 5/6). His brain magnetic resonance imaging was normal. A second lumbar puncture, performed on D30, revealed a high protein level (138 g/dL; normal < 40 g/dL) but no cells. Plasma exchange (PLEX) was started on D57 (1 session every 2 days for 10 days; duration: 2 hours 30 minutes to 2 hours 45 minutes per session; replacement product: 40–50 mL/kg) but without success; his CSF protein level remained high (282 g/dL; normal < 40 g/dL) but there were no cells on D111. In this completely locked-in state, the patient was admitted to our hospital, where on D114 a significant titer of pan-neurofascin (NF155 and NF186) IgG4 autoantibodies (no IgG1, IgG2 or IgG3) was detected. Flow cytometry clearly showed IgG4 anti-pan-neurofascin positivity in human embryonic kidney cells transfected with NF155 and NF186 plasmids and on bead coated with NF155 proteins (Fig. 2). The first rituximab injection was on D124, followed by 20 PLEX sessions administered over 4 months (×5 sessions/month: ×1 session every 2 days for 10 days, every month; duration: 2 hours 30 minutes to 2 hours 45 minutes per session; replacement product: 40–50 mL/kg). However, PLEX was stopped because the patient suffered respiratory distress due to a pulmonary embolism. One month later, on D345, he received the second scheduled administration of rituximab because of the initial severity of the disease and the presence of pan-neurofascin antibodies. Eighty days after the second rituximab injection, the patient’s condition began to improve (D425: GBS-DS score of 4/6). At D532, he was able to do only moderate bilateral stair stepping but he was able to walk 3 km on his own, complaining only of a mild gait disturbance (GBS-DS score: 1/6). An electrophysiological study revealed clear improvement but the CMAP remained low, subsequently attributed to signs of mild demyelination in all 4 limbs (Fig. 1; Table 1). Finally, on D377, his anti-pan-neurofascin autoantibody status became negative and remained negative on D532 (Fig. 2). Rituximab was continued every 6 months for 2 years, resulting in gradual but continuous clinical and electrophysiological improvement.

**Table 1:** Main characteristics of the 6 successive electrophysiological studies of the patient (532 days of follow-up).

					Normal value (N)	EDX1 (day XX) RTX1–X days	EDX2 (day 119) RTX1 −5 days	EDX3 (day 182) RTX1 + 58 days	EDX4 (day 245) RTX1 + 126 days	EDX5 (day 350) RTX1 + 226 days RTX2 + 5 days	EDX6 (day 532) RTX1 + 408 days RTX2 + 187 days
NCS	Motor	Median	Right	Amp (mV)	N > 6		0.4	0.37	1.91	4.7	6.3
				DL (ms)	N < 3.7		19	20.6	9.23	5.58	4.38
				NCV (m/s)*	N > 48		–	–	28.4	39.6	40.2
				CB	No		–	–	−90.1%	No	No
			Left	Amp (mV)	N > 6		0	0	2.6	4.5	4.6
				DL (ms)	N < 3.7		–	–	8.02	5.41	4.15
				NCV (m/s)*	N > 48		–	–	24.8	34.9	41.3
				CB	No		–	–	−79.8%	No	No
		Ulnar	Right	Amp (mV)	N > 6		0	0	1.78	4.5	6.7
				DL (ms)	N < 3.2		–	–	5.59	3.64	2.99
				NCV (m/s)*	N > 50		–	–	19.6	30	38.8
				CB	No		–	–	−97.2%	No	No
			Left	Amp (mV)	N > 6		0	0	2.4	4	ND
				DL (ms)	N < 3.2		–	–	5.5	3.64	ND
				NCV (m/s)*	N > 50		–	–	19.7	35.3	ND
				*CB*	No		–	–	−73.8%	no	ND
		Peroneal	Right	Amp (mV)	N > 3		0	0	0	0	0
				DL (ms)	N < 5		–	–	–	–	–
				NCV (m/s)†	N > 42		–	–	–	–	–
				CB	No		–	–	–	–	–
			Left	Amp (mV)	N > 3		0	0	0	0	0
				DL (ms)	N < 5		–	–	–	–	–
				NCV (m/s)we†	N > 42		–	–	–	–	–
				CB	No		–	–	–	–	–
		Tibial	Right	Amp (mV)	N > 6		0	0	0	0.38	4.7
				DL (ms)	N < 5.5		–	–	–	7.56	4.03
				NCV (m/s)‡	N > 42		–	–	–	31.7	38.6
				CB	No		–	–	–	−64%	No
			Left	Amp (mV)	N > 6		0	0	0	0.53	2.6
				DL (ms)	N < 5.5		–	–	–	6.2	3.81
				NCV (m/s)‡	N > 42		–	–	–	27.6	35.5
				CB	No		–	–	–	No	No
	Sensory	Median (wrist-index)	Right	Amp (µV)	N > 10		0	0	0	10.6	21
				NCV (m/s)	N > 45		–	–	–	37.2	43.4
			Left	Amp (µV)	N > 10		0	0	0	11.6	28.2
				NCV (m/s)	N > 45		–	–	–	33.1	42
		Ulnar	Right	Amp (µV)	N > 8		0	0	0	9	18.4
				NCV (m/s)	N > 45		–	–	–	36.9	46.8
			Left	Amp (µV)	N > 8		0	0	0	5.7	12.7
				NCV (m/s)	N > 45		–	–	–	32.7	52.4
		Superficial peroneal	Right	Amp (µV)	N > 5		0	0	0	0	2.2
				NCV (m/s)	N > 40		–	–	–	–	47.1
			Left	Amp (µV)	N > 5		0	0	0	0	1.67
				NCV (m/s)	N > 40		–	–	–	–	56.4
		Sural	Right	Amp (µV)	N > 10		0	0	0	3.7	9
				NCV (m/s)	N > 42		–	–	–	43.8	64.9
			Left	Amp (µV)	N > 10		0	0	0	5.1	8.9
				NCV (m/s)	N > 42		–	–	–	51.3	49.2
EMG	Spontaneous activity						FiP (++) with rare FaP and PSW in both TA No SA on other muscles	FiP in both deltoids (++), APB (++), in right TA (+++) and in left TA (+) PSW in right TA (++)	FiP in right TA PSW in right TA (++) and left TA (+)	No SA in both left and right TA	No SA in both left and right TA
	Motor unit abnormaities						No muscle contraction in the 4 limbs	No muscle contraction in the 4 limbs	No muscle contraction in right TA Neuropathic pattern in left TA, with low-amplitude MUAPs	Neuropathic pattern in both left and right TA with low-amplitude MUAPs (but higher as in EDX4)	Neuropathic pattern in both left and right TA, with long-duration, high-amplitude, and polyphasic MUAPs

Normal values are in green; abnormal values are in red.

Amp = amplitude, APB = abductor pollicis brevis, CB = conduction block, DL = distal latency, EDX = electrodiagnosis, EMG = electromyography, FaP = fasciculation potentials, FiP =  = fibrillation potentials, µV = microvolts, m/s = meter per second, mV = millivolts, MUAPs = motor unit action potentials, NCS = nerve conduction studies, NCV = nerve conduction velocity, ND = no data, PSW = positive sharp wave, RTX1 = fisrt administration of rituximab, RTX2 = second administration of rituximab, SA = spontaneous activity, TA = tibialis anterior.

* Wrist-elbow.

† Ankle-fibula head.

‡ Ankle-popliteal fossa.

## 3. Discussion

The term autoimmune nodoparanodopathy was first applied to AMAN–AMSAN but later extended to subacute/chronic forms of polyradiculoneuropathy associated with IgG4 antibodies against nodal (neurofascin [NF]-140/186) or paranodal (NF-155, contactin-1/Caspr1) axo-glial proteins. Anti-NF155 and anti-contactin-1 IgG4 are the most frequently detected antibodies, representing 1 to 20% of chronic inflammatory demyelinating polyradiculoneuropathies, with anti-contactin-associated protein-1 and anti-NF140/186 syndromes occurring less frequently. NF-155, contactin-1 and contactin-associated protein-1 attach the terminal myelin loops of the myelinating Schwann cells to the axonal cytoskeleton at the paranode; NF-186 stabilizes NoR, initiates action potentials at the axon initial segment, and contributes to the formation and positioning of the NoR during myelination.^[1]^ In 2017, a shared epitope on the (different) nodo-paranodal neurofascin isoforms 140/155/186 (pan-neurofascin) was identified as a target in acute-onset immune-mediated neuropathy.^[3,4]^ In such cases, the antibodies (mainly of the IgG4 and IgG3 subclasses) directly attack and disturb nodal and paranodal structures, variably associated with complement-mediated effects (in the case of the IgG3/IgG1 subclasses) in the most severely affected patients.

This rare immune-mediated disorder (42 patients reported in the medical literature) triggers a severe form of acute polyradiculoneuropathy that initially mimics GBS but then classically manifests as locked-in syndrome associated with respiratory insufficiency and autonomic instability (Table 1).^[3–16]^ Unlike in GBS patients, an initial intravenous immunoglobulin response is observed in less than half of all patients who, instead, commonly exhibit early and severe relapse, while the response to PLEX or glucocorticoids is usually not sufficient. Immunosuppressive treatments such as rituximab, however, induce remission in most patients (Table 2), but there is no consensus on the treatment regimen and several have proven to be successful (375 mg/m^2^ in 3 weekly doses; 1000 mg with repetition after 2 weeks and subsequent 6-month cycles). Cyclophosphamide, bortezomib, or daratumumab may also yield good results.^[17]^ The present case demonstrates that, although it is obviously preferable to start immunosuppressive therapy as early as possible, later initiation can also be effective.

**Table 2:** Main clinical, biological and therapeutic data of patients affected by pan-neurofascin autoimmune nodoparanodopathy in the medical literature (including our patient).

Reference	Clinical data				Cranial nerve palsy	Biological data	Therapeutic data				Follow-up	
	Number of patients	Average age at diagnosis (yr)	Sex ratio male/female	Mode of onset		NF155 and 140/186 antibodies	IgIV	PLEX	Cortisteroids	Rituximab	Prognosis	Death
Delmont et al, 2017	5	61	3/2	Subacute: 4/5 Chronic: 1/5	3	IgG4: 4 IgG3: 1	4 (FTR: 3)	2 (FTR: 1)	4 (FTR: 3)	1 (FTR: 1)	TI: 5	0/5
Vallat et al, 2018	1	70	1/0	Acute: 1	0	IgG3: 1	1 (FTR: 0)	1 (FTR: 1)	1 (FTR: 1)	0	TI: 1	0/1
Burnor et al, 2018	1	50	1/0	Subacute: 1	1	IgG4: 1	1 (FTR: 0)	1 (FTR:1)	0	1 (FTR: 1)*	TI: 1	0/1
Rempe et al, 2019	1	39	0/1	Chronic: 1	0	IgG (no type)	1 (LTR: 1)	0	1 (LTR: 1)	0	PI: 1	0/1
Stengel et al, 2019	3	69.7	2/1	Acute: 2 Subacute: 1	1	IgG3: 3	ND	ND	ND	ND	ND	ND
Tard et al, 2020	1	76	1/0	Acute: 1	1	IgM: 1	1 (FTR: 0)	1 (FTR: 0)	1 (FTR: 0)	ND	PI: 1	0/1
Li et al, 2020	1	66	1/0	Chronic: 1	0	IgG1: 1	1 (FTR: 0)	1 (LTR: 1)	0	1 (FTR: 1)*	TI: 1	0/1
De Simoni et al, 2020	2	7.9	ND	Chronic: 2	0	IgG4: 2	2 (FTR: 0)	1 (FTR: 0)	1 (FTR: 1)	1 (FTR: 1)	PI: 2	0/2
Fels et al, 2021	1	52	1/0	Subacute: 1	1	IgG4: 1 IgG3: 1	1 (FTR: 0)	1 (FTR: 0)	1 (FTR: 0)	1 (FTR: 1)	PI: 1	0/1
Fehmi et al, 2021	8	68.5	6/2	Acute: 5 Subacute: 3	8	IgG1: 8	8 (FTR: 0)	6 (FTR: 0)	3 (FTR: 0)	4 (FTR: 4)	TI: 1 PI: 3	4/8
Scheibe et al, 2022	1	52	1/0	Acute: 1	1	ND	1 (FTR: 0)	1 (FTR: 0)	1 (FTR: 0)	1 (FTR: 0)	TI: 1 (after daratumumab)	0/1
Wang et al, 2022	1	39	1/0	Chronic: 1	0	IgG4: 1	1 (FTR: 0)	0	1 (FTR: 0)	0	TI: 1	0/1
Appelthauser et al, 2023	11	61.2	9/2	Acute: 4 Subacute: 7	9	IgG3: 11 IgG4: 11	10 (FTR: 6)	11 (FTR: 6)	7 (FTR: 1)	6 (FTR: 6)	PI: 9	2/11
Harris et al, 2023	4	3.75	3/1	Acute: 1 Subacute: 3	0	IgG2: 4	4 (FTR: 2; LTR: 1)	0	3 (FTR: 2; LTR: 1)	1 (FTR: 1)	TI: 4	0/4
Our patient	1	52	1/0	Acute: 1	1	IgG4: 1	1 (FTR: 0)	1 (FTR: 0)	0	1 (FTR: 1)	TI: 1	0/1
Total	42	67	78%/22%	Acute: 38% Subacute: 48% Chronic: 14%	62%	IgG: 97.6% IgG1: 2.5% IgG2: 10% IgG3: 42.5% IgG4: 45% unknown: 2.5% IgM: 2.4%	Used: 95% Response: 35% FTR: 85% LTR: 15% No response: 65%	Used: 68% Response: 37% FTR: 90% LTR: 10% No response: 63%	Used: 60.5% Response: 42% FTR: 75% LTR: 25% No response: 58%	Used: 44,7% Response: 94% FTR: 100% LTR: 0% No response: 6%	Improvement: 85% PI: 51.5% TI: 48.5%	15%

FTR = favorable therapeutic response, LTR = low therapeutic response, ND = no data.

* With cyclophosphamide.

## 4. Conclusion

All GBS/chronic inflammatory demyelinating polyradiculoneuropathy patients presenting with a locked-in state or similar syndrome, not otherwise explained, should be tested for nodo-paranodal pan-neurofascin antibodies. A fulminant immune-mediated pathology, although associated with major disability and a prolonged stay in the intensive care unit, may in fact be curable, albeit sometimes with residual symptoms, if the appropriate immunosuppressive therapy is started as soon as possible.

## Acknowledgments

We thank “TextCheck” for the revision of English. The English in this document has been checked by at least 2 professional editors, both native speakers of English. For a certificate, please see: http://www.textcheck.com/certificate/oLhqHU.

## Author contributions

**Conceptualization:** Elsa Krim, Stéphane Mathis.

**Funding acquisition:** Elsa Krim, Alexandre Masri, Stéphane Mathis.

**Investigation:** Alexandre Masri, Emilien Delmont, Joseph Boucraut.

**Methodology:** Emilien Delmont, Joseph Boucraut.

**Resources:** Gwendal Le Masson.

**Writing – original draft:** Elsa Krim, Stéphane Mathis.

**Writing – review & editing:** Emilien Delmont, Gwendal Le Masson, Joseph Boucraut.
